# Quality Perceptions, Expectations, and Individual Characteristics among Adult Patients Visiting Primary Healthcare Centers in Saudi Arabia: A Cross-Sectional Study

**DOI:** 10.3390/healthcare11020208

**Published:** 2023-01-10

**Authors:** Eidah Alanazi, Hamdah Alanazi, Maha Alanazi, Ahmed Alsadoun, Saeed Asiri, Ghareeb Bahari

**Affiliations:** 1 Ministry of Health, Riyadh 11176, Saudi Arabia; 2 Medical Surgical Department, College of Nursing, King Saud University, Riyadh 11421, Saudi Arabia; 3 Nursing Administration and Education Department, College of Nursing, King Saud University, Riyadh 11421, Saudi Arabia

**Keywords:** primary healthcare centers, patient perceptions, expectation, service quality

## Abstract

Quality is a main concern of primary healthcare centers, and pursuing quality can lead to service improvement as well as affordable healthcare. The purpose of this cross-sectional study was to describe patients’ healthcare quality perceptions and expectations and determine the relationships between them and associated factors. The study was conducted on a convenience sample of 470 patients visiting primary healthcare centers. Data were collected between April and July 2022 using an anonymous questionnaire. Bivariate and multivariate analyses were conducted. Most participants reported high levels of quality perceptions and expectations. Bivariate analyses showed a significant correlation between quality perceptions and expectations. Both being single and having a higher level of education were statistically different in terms of quality perception and expectations, respectively. Further, being single, highly educated, and employed had significant differences in terms of expectations. In regression, primary education and expectations influenced quality perceptions. Marital status, profession, and perception were the only variables that significantly influenced participants’ expectations. Patients’ healthcare quality perceptions and expectations are important for ensuring the efficiency of healthcare services. Primary healthcare centers are the key avenue for disease prevention and early detection. The optimization of primary healthcare centers’ quality and addressing its potential issues should be performed through interdisciplinary teamwork.

## 1. Introduction

Primary healthcare centers are the foundation of any country’s healthcare system as they serve as the primary access point to healthcare systems, treatment, disease prevention, and improved quality of life [1]. The key requirements for primary healthcare centers include them being effective in terms of costs and procedures, access to the public, and acceptable to the community they serve [2]. High quality, integrated, and safe practices should be provided by health professionals who are skilled, committed to their work, well trained, and motivated by respect [3]. Further, primary healthcare centers can also improve patients’ safety by reducing medical errors, enhancing patients’ quality of life, and improving staff performance [1]. In Saudi Arabia, primary healthcare centers are a nationwide system managed by the Ministry of Health to provide free healthcare services to Saudi citizens living with non-communicable diseases or who need simple procedures. More than two thousand healthcare centers are located in the thirteen regions of the country to make them accessible to citizens who live near them. The Ministry seeks, in its strategic objectives, to improve the quality of health services provided in these centers, as well as other health facilities, in order to meet patients’ various needs and ensure their safety. Primary healthcare centers are staffed by health professionals such as physicians, nurses, pharmacists, and laboratory specialists. Such centers can be led by physicians or nurses. Simple equipment is also used to provide primary health care for the least invasive cases. For cases with high risk, they are transferred to hospitals.

Quality is a main concern of primary healthcare centers, and pursuing quality can lead to service improvement, as well as affordable healthcare [4]. Offering good quality services is of crucial importance in the management of service companies. Good quality can also contribute to reducing risks to patients’ safety [1]. Medical facilities, in particular, seek to provide outstanding clinical care and quality services to their patients. Primary healthcare centers’ quality depends not only on patients’ experiences but also on staff members’ practices, responsibility, and creativity. Therefore, improving quality should be considered an integral part of primary healthcare centers’ working environments [5].

Perception refers to how services are perceived and how they are seen, heard, or felt. Traditionally, healthcare systems follow health standards that help ensure patient care quality [6]. Organizations that prioritize consumers and approach problems from the customers’ perceptions are more successful in today’s unpredictable and competitive market [7]. Patients’ willingness to use goods and services in the future is influenced by perceived quality, as this is a primary factor that influences consumer satisfaction [8]. The success of the implementation of any healthcare delivery system, including telemedicine, depends heavily on patients’ perceptions. The primary source of information for these perceptions is the patients who provide their opinions on whether adequate medical care is being provided and if the medical care they receive meets their expectations [9].

Expectations in healthcare refer to the attitudes or beliefs in terms of what is available in a counseling service or healthcare system; it is a mental picture that the general public has for the process of interacting with the healthcare system [10]. Service quality is simply the services’ ability to meet consumers’ expectations. In other words, patients’ expectations of health services quality would be better if such services were readily available [11]. Healthcare facilities aiming to support patients’ expectations of improved quality could influence healthcare and the healthcare services’ adherence and compliance to policies aimed at improving quality [12]. Patients’ expectations need to be considered in healthcare service delivery as this information is effective not only for experts but also for achieving service quality objectives.

In some countries, such as Saudi Arabia, there has been a significant improvement in healthcare services in order to meet the requirements caused by urbanization and lifestyle changes. In 2020, there were about 2257 primary healthcare centers available to meet Saudis’ healthcare needs. This number is expected to rise as it is one of the goals of the national transformational plan called Vision 2030. Primary healthcare centers, in addition to other health settings, could improve the quality and efficiency of healthcare services. However, the role of primary healthcare centers is relatively weak and unsupported. Based on that, this study was conducted to describe patients’ healthcare quality perceptions and expectations and determine the relationships between them and associated factors. Findings would provide helpful knowledge on improving quality to policy makers.

## 2. Materials and Methods

### 2.1. Study Design and Sampling

This research was a cross-sectional study conducted on a convenience sample of patients visiting primary healthcare centers. The sample comprised Saudi adult patients who visited primary healthcare centers seeking treatment for common illnesses such as diabetes. Patients who were willing to complete the survey also participated in the study. Patients who were unable to read or understand the survey items were excluded from participation. Those who did not wish to participate were also excluded. Respondents were recruited from three primary healthcare centers that were easily accessible to researchers. Because of the population density in Riyadh (approximately 7 million), there are around 430 primary healthcare centers managed by the Ministry of Health. To determine the minimum sample size for the study, we used the Raosoft online sample calculator. With a margin of error of 5%, a confidence interval of 95%, a response distribution of 50%, and a population of approximately 6000, the recommended sample size was 362 subjects. As missing data are common in research, this sample size was increased by 20%, which led to a minimum required sample size of 434.

### 2.2. Ethical Consideration

Ethical approval was obtained from the institutional review board located at King Fahad Medical City (Reference #: 22-007E). Informed consent was ensured from all participants. They were also informed that their participation was voluntary, and they had the right to withdraw from the study at any time without penalty.

### 2.3. Measuring Instruments

In addition to the demographic questions, which included age, nationality, gender, social status, education, and occupation, we used SERVQUAL to measure service quality [13]. This 5-point Likert scale included 44 items that measured two subscales: perceptions and expectations [14]. Each subscale included five dimensions: reliability, responsiveness, assurance, empathy, and tangibility. The shortened Arabic-validated version of the scale (M., Ishfaq, personal communication, 12 September 2019), which included 40 items, was used as Arabic is the official language of Saudi Arabia.

#### 2.3.1. Perceptions

This subscale included 20 items that measured consumers’ quality perceptions of current services. The questions were distributed to address the five dimensions, with reliability being represented in 5 items, responsiveness in 4 items, assurance in 4 items, empathy in 3 items, and tangibility in 4 items. The answers ranged from 1 (strongly disagree) to 5 (strongly agree), with a higher total score (100) indicating good perceptions of quality. The Cronbach’s alpha for the perception subscale was 0.97.

#### 2.3.2. Expectations

This subscale included 20 items that evaluated consumers’ expectations of better quality in the future. The questions were distributed to address the 5 dimensions, with reliability being represented in 5 items, responsiveness in 4 items, assurance in 4 items, empathy in 3 items, and tangibility in 4 items. The answers ranged from 1 (strongly disagree) to 5 (strongly agree), and higher scores (above 100) indicated better quality expectations. The Cronbach’s alpha for the expectation subscale was 0.98.

### 2.4. Data Collection Procedures

Data collection spanned April and July 2022. Several recruitment strategies, including posting flyers on bulletin boards, word of mouth, and personal referrals, were used to facilitate data collection. After agreeing to participate, the participants were provided with the questionnaire form that explained the study’s purpose and procedures. Eligible participants had the option to complete the survey in the waiting room and without help from a professional or at home and return the form upon completion.

### 2.5. Data Analysis

Data were analyzed using SPSS (V.28, IBM Corp., Armonk, NY, USA); descriptive statistics, including frequencies, percentages, means, and standard deviations (SDs), were calculated. Frequency distributions also helped screen for missing data, which provided no more than 2% of data were missing per item. The mean imputation method was used to handle missing data [15]. The Kolmogorov–Smirnov test was run to show whether data were normally distributed with a value of 95% confidence. A histogram is another method used to test the normality of the continuous data, which showed no major violation. The level of education was changed into binary categories for further analysis. An independent samples *t*-test, Pearson’s correlation coefficient (r), and multiple linear regression were all used where needed. The variable “level of education” was combined to create two dummy variables for regression. The statistical significance was set at a level of 0.05.

## 3. Results

### 3.1. Sample Characteristics

Table 1 provides the sample’s characteristics (N = 470). The participants’ mean age was 33.78 (SD = 7.24 years; range = 20–60). The majority of the sample was male (75.7%) and not married (66.4%). Almost more than three-quarters of the sample were employed (89%). Further, most of the sample held a high school degree (49.1%). Most participants reported high quality perceptions (M = 79.57; SD = 19.9; range = 20–100) and expectations (M = 83.68; SD = 18.56; range = 20–100).

### 3.2. Bivariate and Multivariate Analyses

Bivariate analyses are presented in Table 2. In the independent samples *t*-test, there were significant differences between marital status and level of education in terms of quality perception (t208.845 = −4.490; *p* < 0.001) and expectations (t362.647 = −3.586; *p* < 0.001), respectively. Further, marital status (t203.153 = −5.302; *p* < 0.01), level of education (t389.087 = −2.197; *p* < 0.05), and employment status (t60.067 = 2.608; *p* < 0.05) had significant differences in terms of expectations. When running Pearson’s correlation coefficient, age was found to be significantly negatively associated with perception (r = −0.130; *p* < 0.001). There was also a significant positive correlation between quality perception and expectations (r = 0.744; *p* < 0.001). In the regression analyses, the quality perception model was significant (*F* (7,469) = 86.270; *p* < 0.05; R^2^ = 0.567) (see Table 3). Among the variables, only primary education (B = −3.584; *p* = 0.009) and quality expectations (B = 0.790; *p* < 0.001) showed significant associations with perceptions. The quality expectations model was also significant (*F* (7,469) = 89.436; *p* < 0.05; R^2^ = 0.575) (see Table 4). Among the variables in the model, only marital status (B = 5.205; *p* < 0.05), employment status (B = −3.979; *p* < 0.05), and perception (B = 0.674; *p* < 0.05) statistically influenced expectations.

## 4. Discussion

This study identified patients’ quality perceptions and expectations of healthcare services and associated factors. Even though participants’ perceptions of the quality of healthcare services were high, they still had higher expectations for more and various healthcare services. This is similar to the findings of a recent study conducted in Jordan in which the differences between patients’ perceptions and expectations of healthcare services quality were examined, which concluded that patients’ expectations were higher than their perceptions [16]. In Iran, researchers evaluated the quality of health services in Ahvaz healthcare centers using SERVQUAL [7]. They found higher expectations in terms of the quality of healthcare services compared to the participants’ perceptions. Similarly, some studies in Romania and Malaysia had results that are consistent with our study’s findings [17,18]. Based on Vision 2030, the government seeks to improve the quality of primary healthcare centers’ health services.

In the bivariate analyses, there were significant differences in the participants’ quality perceptions when they had different levels of education and marital status. There were also significant differences in the participants’ expectations according to these two variables in addition to the participants’ employment statuses in that the participants with higher educational levels perceived and expected better quality healthcare. The authors reported no significant association between educational status and patients’ perceptions [10], while another study reported education level to be correlated with expectations [19]. The reason for the difference in findings between our study and Girmay et al.’s [10] may be due to the study location, as the current study targeted primary healthcare centers, while the other study was conducted on patients who visited hospitals for treatment. Regarding marital status, single people usually engage in riskier health-related activities which require health treatment [20]. In addition, a cross-sectional study stated that perceptions of employees with mental health symptoms who struggle with work are strongly correlated with their expectations [21]. This study, however, emphasized employees’ work perceptions and return-to-work expectations only. There is little research on perceptions and expectations of health services quality provided to employees. Employed people likely have higher quality expectations as they likely know about healthcare systems due to periodic health assessments that are mandated by their companies and organizations.

Additional bivariate findings showed that age was found to be significantly negatively correlated with patients’ quality perceptions of the primary healthcare centers’ services. Adults and the elderly might experience worse healthcare services at primary healthcare centers. Similarly, age was reported negatively correlated with quality perceptions [19]. Elderly people usually had lower quality perceptions of healthcare services [22]. In contrast, another study found that age had no significant relationship with quality perception [16]. The differing results may be due to the outbreak of COVID-19 and the resulting lockdown imposed by the government. COVID-19 preventive measures, including confinement, reduced the quality of care services for patients with chronic conditions [23]. In the bivariate analyses, the participants’ quality perceptions were also found to have a strong positive correlation with their quality expectations. Participants who had good perceptions of a primary healthcare center’s quality had positive expectations of better services in the future. This finding is similar to the findings of a prior study [10]. However, the lack of quality studies on this topic in Saudi Arabia is still a challenge. Another study conducted on patients visiting primary healthcare centers in Jubail, Saudi Arabia, [24] found that patients mostly fluctuated positively towards the level of provided care. Though the authors provided important information on patients’ satisfaction with primary healthcare centers’ services, it may be difficult to generalize their findings beyond their sample. Their study was conducted in a relatively small governorate with limited public and health services. The current study was implemented in the city of Riyadh, where approximately 8 million people live, and greater improvements are available in health services since it is the capital of the country. Further, their study was performed on a sample that lives in a specific compound, unlike the current study, which was conducted at three public primary healthcare centers. Based on that, more research that compares patients’ perceptions and expectations of hospitals and primary care centers’ services in the Saudi context are needed.

Similar to the bivariate analyses, the multivariate analysis revealed that primary education influenced quality perceptions. The multivariate analysis also reported that only marital status, employment status, and quality perceptions influenced patients’ expectations of primary healthcare centers’ quality. This was in line with previous studies [25,26,27]. However, in a prior study, there were inconsistent findings [28] where authors reported that marital status was not significantly associated with the assessment of primary care centers’ quality. The performance of healthcare providers could be enhanced by monitoring patients’ perceptions of healthcare services [29]. Healthcare professionals’ decisions about primary healthcare centers’ quality are important as they will allow for the provision of high-quality services to patients. The balance between technical quality and patients’ perceptions and expectations of healthcare services should be considered in order to ensure better healthcare quality at primary healthcare centers. Further, as the incidence of chronic conditions is on the rise due to lifestyle changes [30], good healthcare services are needed.

### 4.1. Study Limitations

There are some limitations to be noted. Firstly, there may be bias resulting from the convenience sampling strategy. Secondly, a cross-sectional study design cannot evaluate cause-and-effect relationships in the same way as a longitudinal study design. Thirdly, the data were collected from three primary healthcare centers located in one region and within the same healthcare system, which may result in difficulties in generalizing the results beyond the study’s sample. The differences between them to compare each primary healthcare center’s quality were also not determined. In addition, the outbreak of COVID-19 may have influenced the participants’ responses since they did not visit primary healthcare centers for almost two years because of the lockdown. Though the age group and marital status are similar to national indicators, the homogeneity of the sample may be another limitation, specifically that participants were educated, employed, and married males. Findings may not be generalized to Saudi women, as well as those who are illiterate or with limited health literacy. Despite these limitations, information that can contribute to improving the quality of health services at primary healthcare centers was provided.

### 4.2. Study Implications

This study has important implications for clinical practice. For example, marital status was correlated with both the perceptions and expectations of primary healthcare centers’ quality. Therefore, healthcare professionals should improve health services that support individual and family health, and they should provide awareness programs to help meet public health needs. Participants with higher education levels perceived and expected better quality at primary healthcare centers. Based on this, healthcare decision makers in primary healthcare centers should consider patients’ levels of education in order to improve patients’ understanding of the treatment plan. For example, they could create helpful activities that explain healthy behaviors to patients because patients with low education levels will likely be unable to understand treatment plans. More research is needed to investigate the ways in which to enhance patients’ perceptions and expectations of primary healthcare centers’ quality and how this pursuit can be affected by healthcare professionals.

It is a fact that employed people had higher quality expectations than unemployed people. Therefore, healthcare providers should improve the health services provided to patients, particularly those who are unemployed. Further, policy makers should bear in mind that employees may be aware of quality standards as they may be involved in quality standards in their workplace. This study also indicates that patients’ quality perceptions and expectations were correlated. Therefore, meeting patients’ health needs to ensure better healthcare services is necessary. Healthcare providers also have to make every effort to increase the positive perceptions and expectations of quality.

## 5. Conclusions

A positive correlation was found between healthcare quality perceptions and expectations in primary healthcare centers. Primary education influenced patients’ perceptions of primary healthcare centers’ quality. Further, being single, highly educated, employed, and having quality perceptions influenced patients’ expectations of better quality. Patients’ healthcare quality perceptions and expectations are important for ensuring the efficiency of healthcare services. As a result, policy makers at the Saudi Ministry of Health should understand and integrate patients’ perceptions and expectations into service plans so as to ensure positive outcomes in primary healthcare centers. Future studies that address the potential issues in quality perceptions and expectations in primary healthcare centers are also recommended.

## Figures and Tables

**Table 1:** Sample characteristics (N = 470).

Characteristics	n	(%)
Age (Years) M = 33.78, SD = 7.28, range: 20–60		
20–30	164	(34.9)
31–45	274	(58.3)
>45	28	(6)
**Gender**		
Male	356	(75.7)
Female	114	(24.3)
**Marital status**		
Not married	312	(66.4)
Married	158	(33.6)
**Level of education**		
Elementary and/or intermediate (primary)	221	(47)
High school	231	(49.1)
College level	18	(3.8)
**Employment status**		
Employed	418	(88.9)
Unemployed	52	(11.1)

Note: M = Mean; SD = standard deviation.

**Table 2:** Mean differences between perception, expectations, and some demographics.

Variable Mean Differences (*t*-Test)	Binary Categories	Perception *M* (*SD*)		*p*-Value	Expectation *M* (*SD*)		*p*-Value
Gender	Male Female	78.83 81.87	18.93 22.59	0.351	83.39 84.57	17.34 22.03	0.098
Marital status	Married Not-married	72.88 82.95	26.16 14.74	˂0.001 *	76.30 87.41	24.63 13.11	˂0.001 *
Level of education **	Intermediate or less High school or more	76.03 82.71	23.80 15.01	˂0.001 *	81.65 85.48	21.60 15.19	˂0.001 *
Employment status	Employed Unemployed	80.16 74.78	19.53 22.26	0.301	84.58 76.43	17.97 21.65	0.035 *

Note. * *p*-value < 0.05; ** Level of education data were joined into binary categories.

**Table 3:** Multiple regression analysis for variables associated with perception.

Variables	Unstandardized Coefficients		Standardized Coefficients	Sig.	Model Summary	
	B	Std. Error	Beta		R^2^	*p*-Value
(Constant)	18.237	6.730		0.007	0.567	˂0.001 *
Age	−0.139	0.092	−0.051	0.129		
Gender	1.179	1.505	0.025	0.434		
Marital status	−0.669	1.492	−0.016	0.654		
Primary Education	−3.584	1.365	−0.090	0.009 *		
College Education	−1.023	3.318	−0.010	0.758		
Employment status	1.188	2.087	0.019	0.569		
expectations	0.790	0.034	0.737	<0.001		

Note: High school is reference for primary and college education variables; Being single is reference for marital status variable; employed is reference for employment status variable * *p*-value < 0.05.

**Table 4:** Multiple regression analysis for variables associated with expectation.

Variables	Unstandardized Coefficients		Standardized Coefficients	Sig.	Model Summary	
	B	Std. Error	Beta		R^2^	*p*-Value
(Constant)	30.125	6.008		˂0.001	0.575	˂0.001 *
Age	0.037	0.085	0.015	0.659		
Gender	−1.597	1.389	−0.037	0.251		
Marital status	5.205	1.357	0.133	˂0.001 *		
Primary Education	2.362	1.265	0.64	0.063		
College Education	1.003	3.064	0.010	0.744		
Employment status	−3.979	1.919	−0.067	0.039 *		
Perception	0.674	0.029	0.722	˂0.001 *		

Note: High school is reference for primary and college education variables; Being single is reference for marital status variable; employed is reference for employment status variable * *p*-value < 0.05.

## Data Availability

Data are not shared due to privacy and ethical restrictions.
